# Hypophosphatemic Rickets in Siblings: A Rare Case Report

**DOI:** 10.1155/2016/4803167

**Published:** 2016-05-31

**Authors:** Gummadapu Sarat, Nuthalapati Priyanka, Meka Purna Venkata Prabhat, Chintamaneni Raja Lakshmi, Sujana Mulk Bhavana, Dharmavaram Ayesha Thabusum

**Affiliations:** Department of Oral Medicine & Radiology, Drs. Sudha and Nageswara Siddhartha Institute of Dental Sciences, Chinoutpalli, Gannavaram Mandal, Andhra Pradesh 521286, India

## Abstract

Hypophosphatemic rickets (HR) is a type of hereditary rickets characterized by persistent hypophosphatemia and hyperphosphaturia. The most predominant type is inherited in an X-linked fashion and caused by mutation in the gene encoding the phosphate-regulating endopeptidase homolog, X-linked (PHEX), identified in 1995. The X-linked hypophosphatemic (XLH) rickets is a rare hereditary metabolic disorder with a prevalence of 1 in 20,000 and causes deficient calcification of mineralized structures such as bones and teeth resulting in dental problems in terms of delayed eruption, spontaneous periapical infections, and exfoliation. We report one such unique case of hypophosphatemic vitamin D-resistant rickets in siblings exhibiting classical features of rickets with emphasis on its treatment and dental considerations.

## 1. Introduction

Rickets, a disease that occurs in children, is the failure of mineralization of osteoid commonly associated with either vitamin D deficiency or decrease of serum phosphate levels leading to hypophosphatemia. The condition, hypophosphatemic rickets, first described by Albright et al. [1] in 1937, with a prevalence of 1 in 20,000 [2], may be hereditary or acquired. Among these familial hypophosphatemia conditions is a rare inherited disorder [3]. It is most often inherited as X-linked trait with dominant transmission; however autosomal dominant and recessive forms also occur [4].

The case report describes the clinical, radiological, and biochemical features of hypophosphatemic rickets in siblings who came to the clinic with a chief complaint of missing teeth.

## 2. Case Report

A 10-year-old girl (Case 1) presented to the department of oral medicine with a chief complaint of missing teeth in the upper front region of the jaw for 7 years. There was history of trauma to the upper front region of jaw at about 3 years of age resulting in loss of deciduous teeth and delay in the eruption of permanent teeth.

### 2.1. Case 1

The patient is the second offspring after a full term normal uneventful pregnancy.

**Figure 1:**
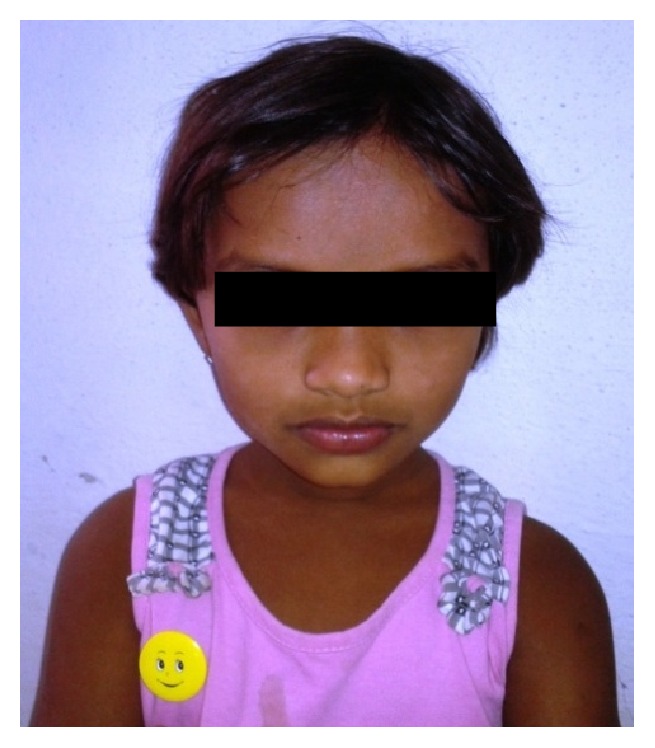
Showing frontal bossing and depressed nasal bridge.

**Figure 2:**
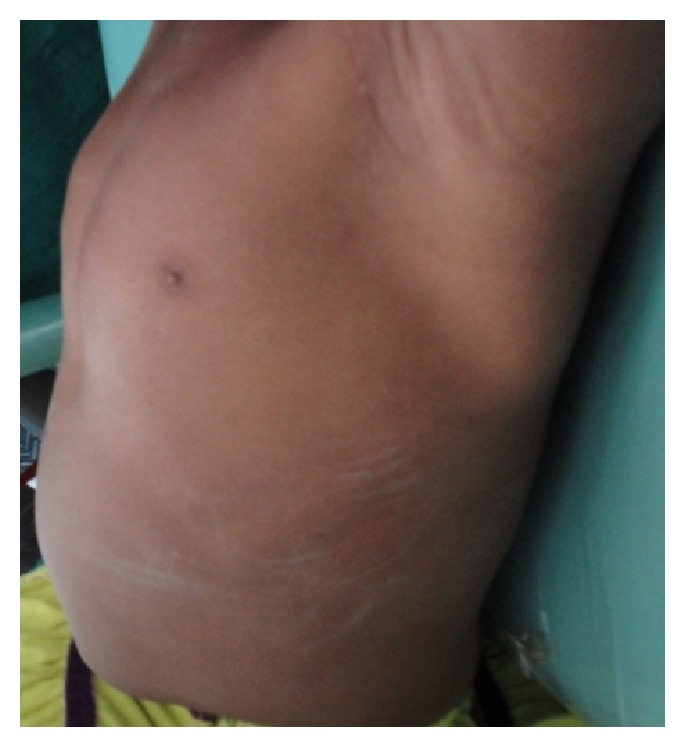
Showing no signs of rachitic rosary and Harrison's groove.

**Figure 3:**
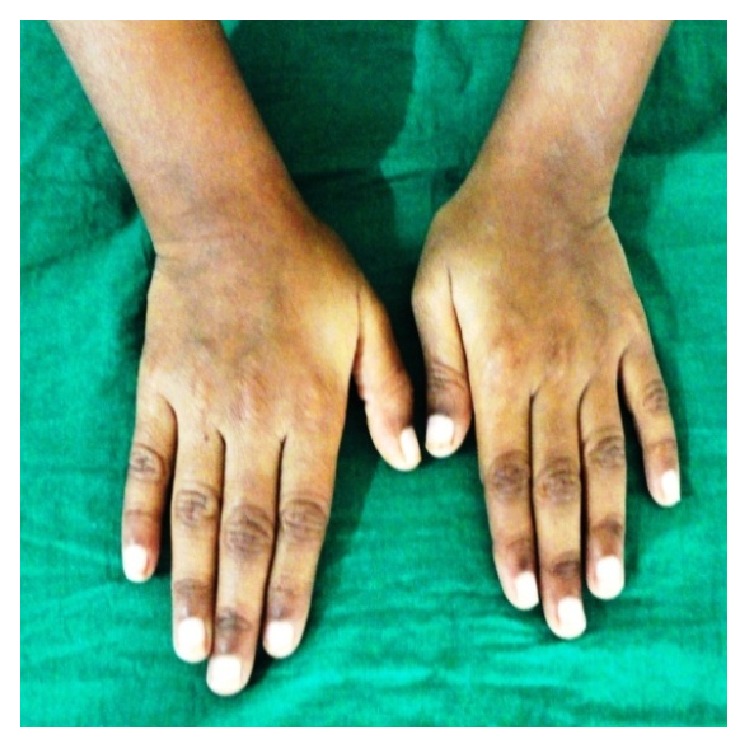
Showing no signs of widening of metaphysis.

**Figure 4:**
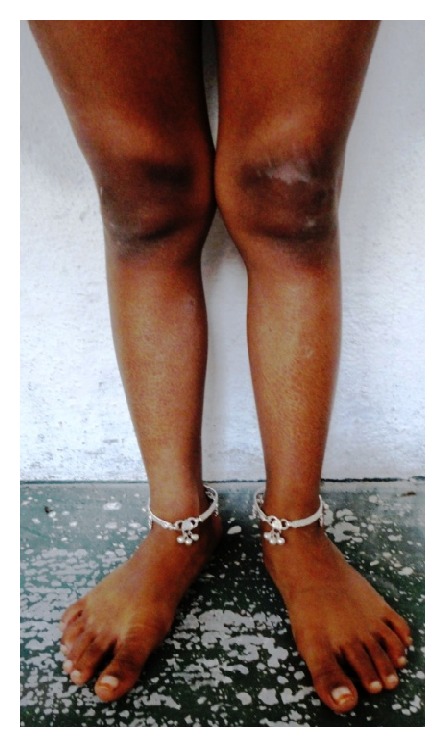
Showing knock knees.

*General examination* (Case 1) revealed the height and weight as 45 inches and 14 kgs, which are less when compared with average height and weight of an age-matched Indian girl (average height: 54.4 inches, average weight: 32.5 kgs). There were frontal bossing, depressed nasal bridge (Figure 1), and knock knees (Figure 3). No signs of rachitic rosary (Figure 2), enlargement of wrist (Figure 3) and ankle joints, and bowed legs (Figure 4) were observed.

**Figure 5:**
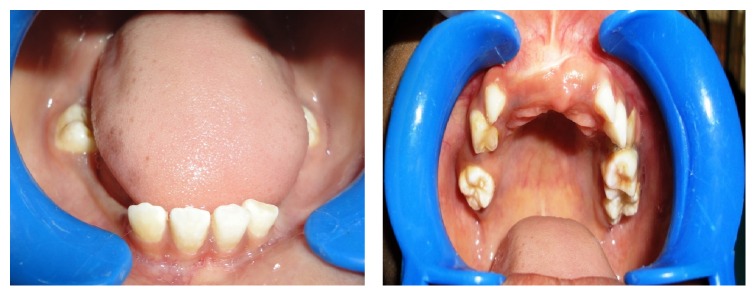
Showing missing teeth in relation to upper and lower anterior ends and mixed dentition.

*On intraoral examination*, caries-free mixed dentition was present with missing permanent maxillary centrals and laterals with fair oral hygiene (Figure 5).

*Radiographic Findings.* Chest radiograph, radiograph of hip with femur and tibia, wrist radiograph, and orthopantomograph (OPG) were advised.

Radiographic findings of chest radiograph, radiograph of hip with femur and tibia, and wrist radiograph were interpreted by a general radiologist with 30-year experience.

**Figure 6:**
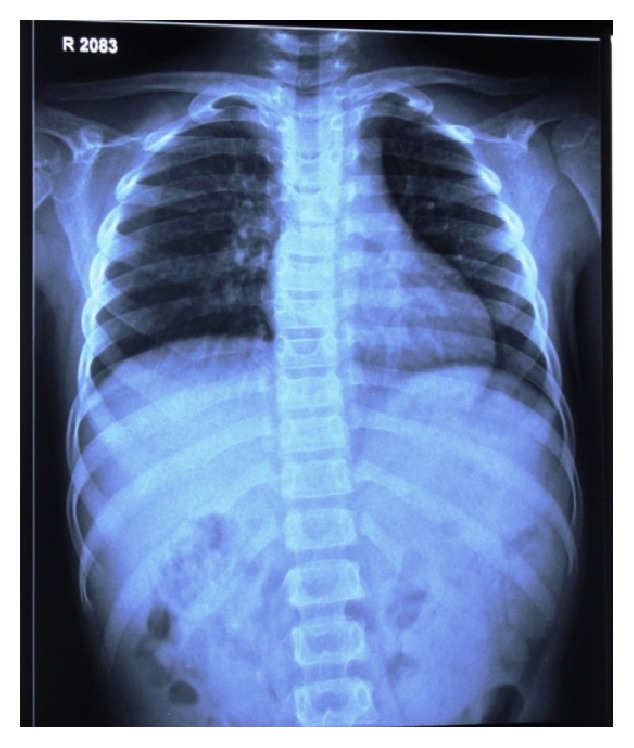
Chest radiograph.

**Figure 7:**
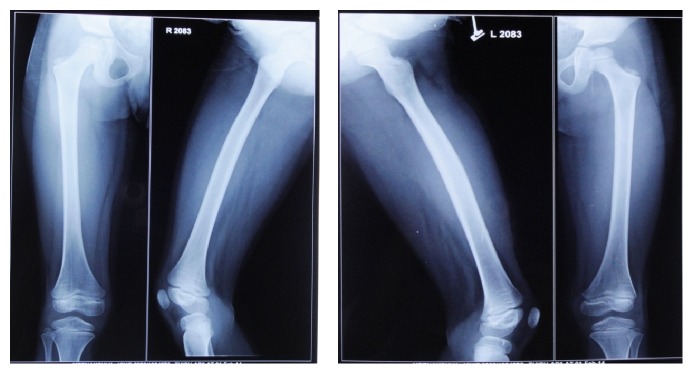
Radiograph of hip with femur and tibia.

**Figure 8:**
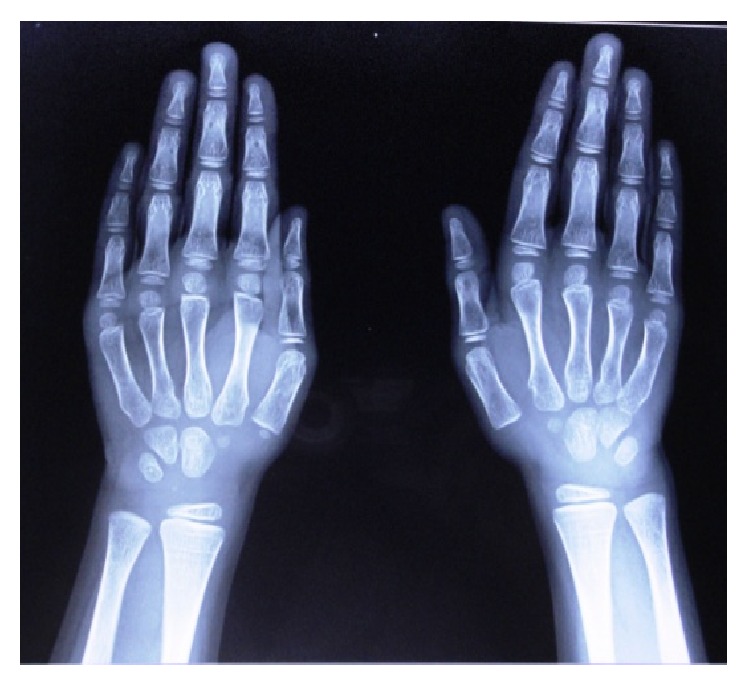
Wrist radiograph.

**Figure 9:**
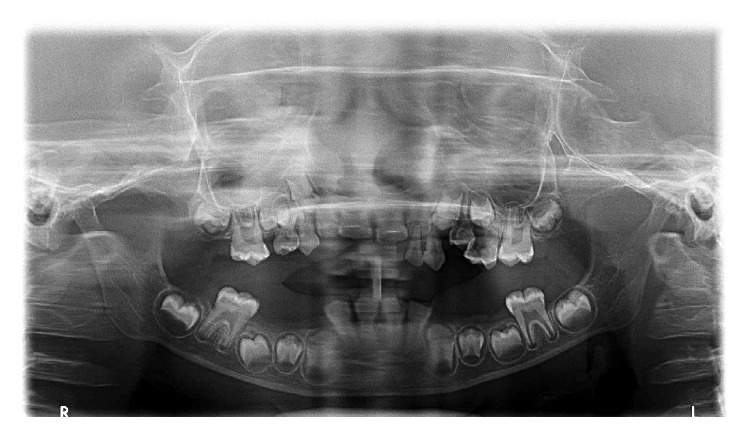
OPG.

1. Chest radiograph (Figure 6) revealed mild scoliosis.
2. Radiograph of hip with femur and tibia (Figure 7) revealed coxavara deformity.
3. Wrist radiograph (Figure 8) revealed (a) epiphysis for radius noted but epiphysis for ulna not yet visualised, (b) few growth arrest lines noted in the lower end of radius, and (c) skeletal age found to be 5 yrs.
4. OPG (Figure 9) revealed (a) the presence of deciduous teeth 53, 54, 62, 63, and 65, (b) presence of all the permanent teeth, and (c) enlarged pulp chambers with highly placed pulp horns extending to the dentinoenamel junction.

### 2.2. Case 2

On further investigation, the patient's mother revealed history of consanguineous marriage and that her younger daughter (Case 2), who is 7 years old, also showed similar features.

**Figure 10:**
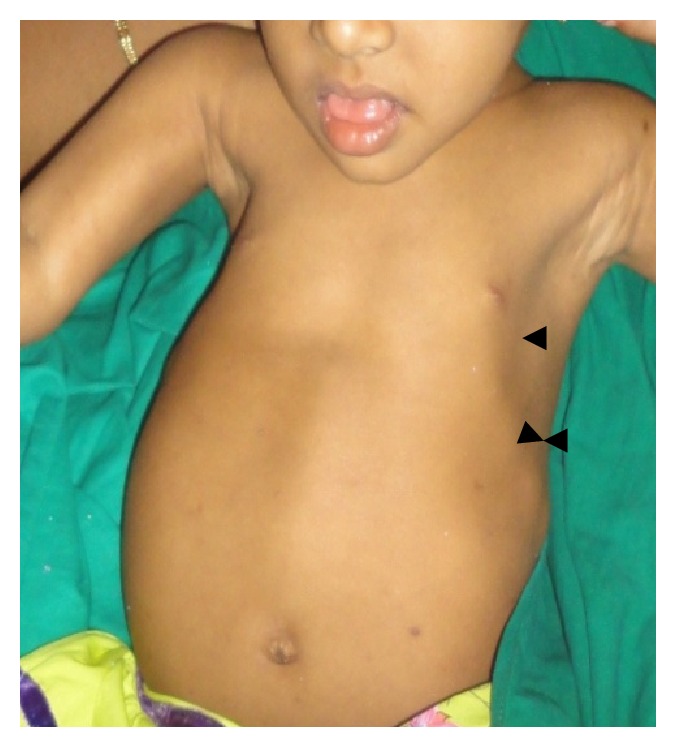
Showing rachitic rosary, incompetent lips, and macroglossia.

**Figure 11:**
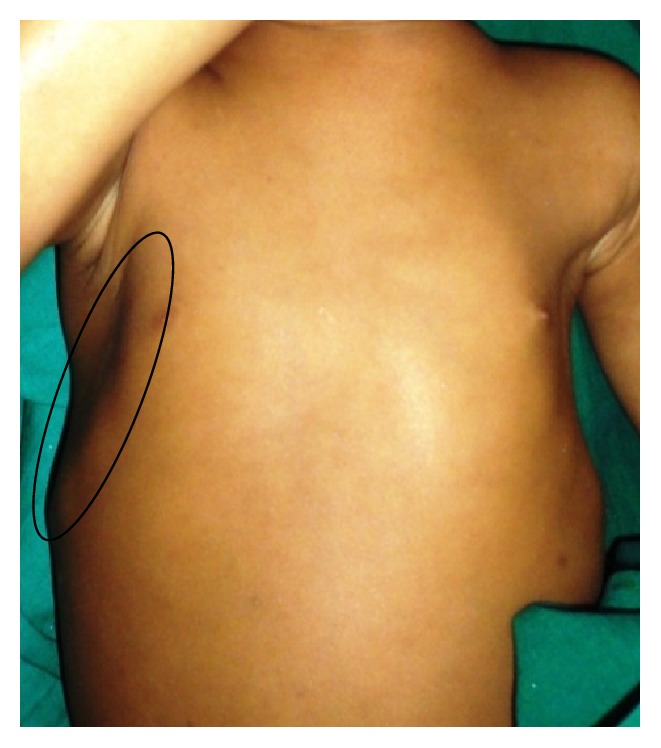
Showing Harrison's groove on the chest region.

**Figure 12:**
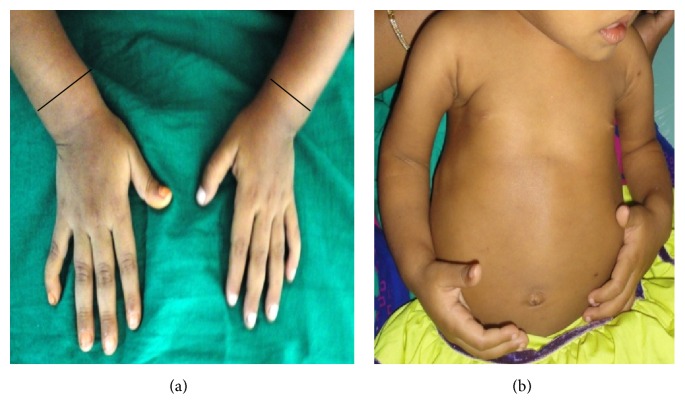
Showing widening of metaphysis.

**Figure 13:**
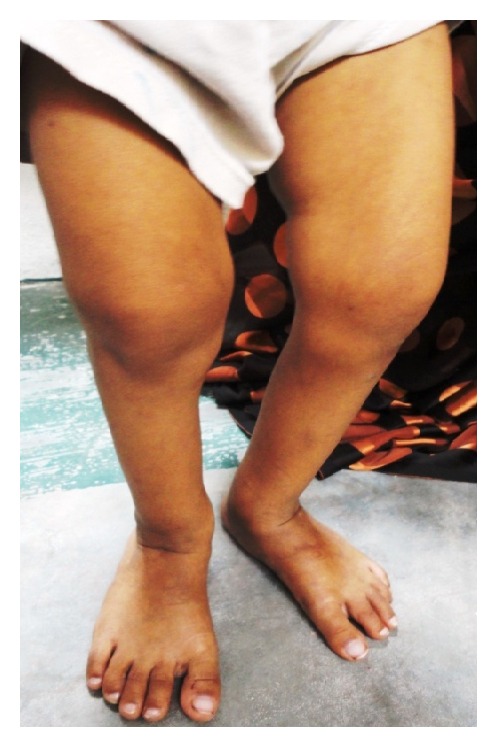
Showing bowed legs and enlargement of ankles.

*General examination* revealed the height and weight as 23 inches and 10 kgs, which are less when compared with average height and weight of an age-matched Indian girl (average height: 47.4 inches, average weight: 21.8 kgs). Further examination revealed the clinical characteristics of base diagnosis given: There was bowing of legs (Figure 13) and enlargement at the wrist (Figure 12) and ankle joints (Figure 13). The presence of rachitic rosary (Figure 10) and Harrison's groove (Figure 11) was also noted.

**Figure 14:**
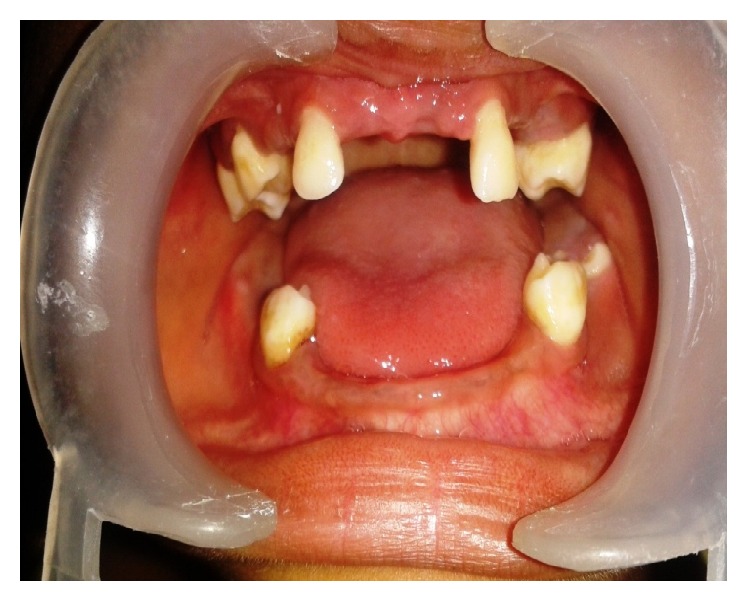
Showing mixed dentition and missing teeth in relation to upper and lower anterior ends.

*On intraoral examination*, incompetent lips, macroglossia (Figure 10), and caries-free mixed dentition were present with missing maxillary centrals, canines, and mandibular lateral incisors with fair oral hygiene (Figure 14).

*Radiographic Findings.* Chest radiograph, radiograph of hip with femur and tibia, wrist radiograph, and intraoral periapical (IOPA'S) radiographs were advised.

**Figure 15:**
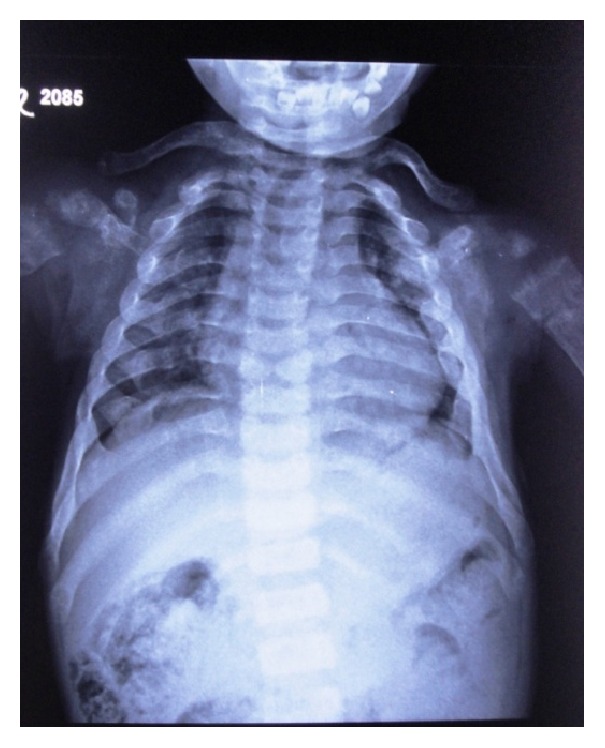
Chest radiograph.

**Figure 16:**
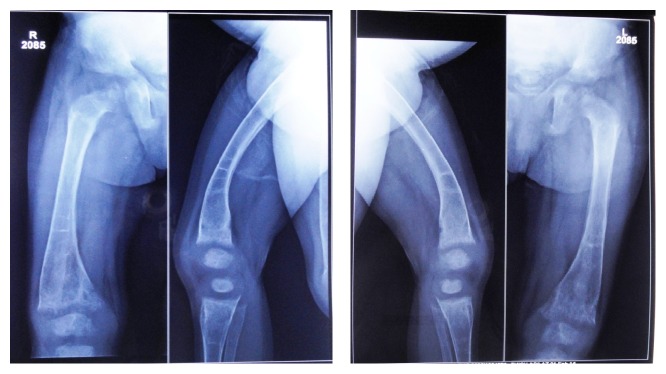
Radiograph of hip with femur and tibia.

**Figure 17:**
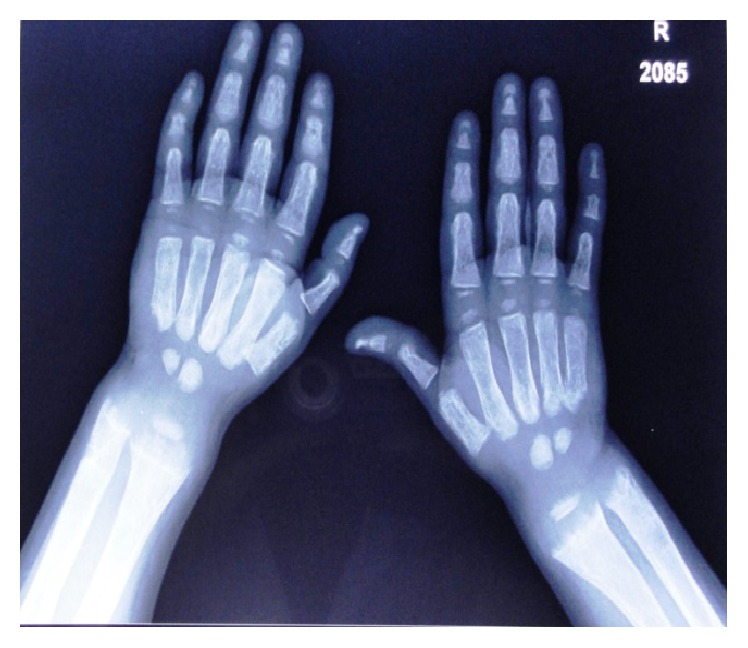
Wrist radiograph.

**Figure 18:**
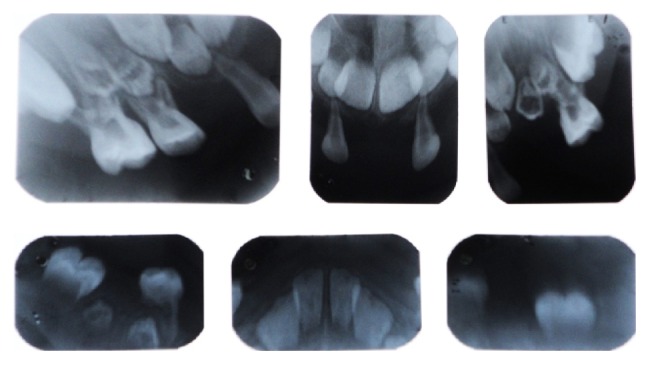
Intraoral periapical radiographs.

1. Chest radiograph (Figure 15) revealed (a) mild scoliosis, (b) both humeral metaphyses showing fraying of bones with resorption in their proximal ends, and (c) ribs being broadened and anterior ends of ribs appearing to be widened which is the classical feature of rickets.
2. Radiograph of hip with femur and tibia (Figure 16) revealed (a) cupping/fraying in both lower end of femur and upper end of tibia in both hips, (b) cortical thinning, (c) bowing of legs, (d) growth arrest lines, (e) epiphyseal widening, and (f) femoral ossific nuclei not fully developed and appearing to be superiorly migrated.
3. Wrist radiograph (Figure 17) revealed (a) widening and cupping/fraying of metaphysis in the lower ends of radius and ulna, (b) significant osteoporosis, (c) epiphysis for radius visible but epiphysis for ulna not yet visualised, (d) evidence of periostitis in metacarpals, (e) resorption of bones seen, and (f) skeletal age found to be 2 yrs.
4. IOPA'S (Figure 18) shows the presence of deciduous teeth 52, 54, 55, 62, 65, 74, and 84 and all the permanent teeth.

*Laboratory investigations* of both Cases 1 and 2 are presented in Table 1. The ultrasound report of kidneys revealed early renal disease and complete blood picture revealed leucocytosis and thrombocytosis in both siblings.

## 3. Treatment

**Figure 19:**
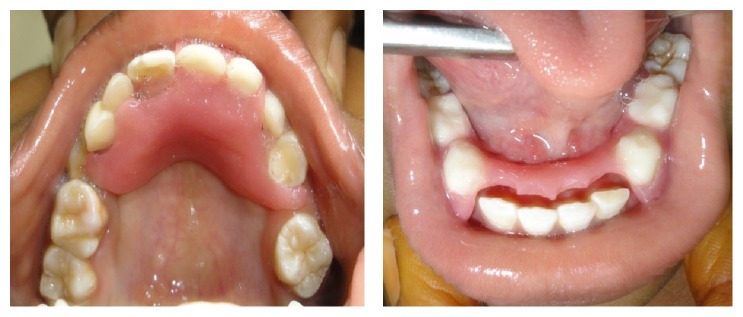


The general management is taking care by using neutral phosphate and calcitriol supplements for both siblings. As the patient's chief complaint was delayed eruption of teeth, Case 1 was given denture (Figure 19) so that it causes a stimulating effect on the underlying permanent teeth and causes its eruption along with fulfillment of esthetics. Case 2 was kept under observation for eruption of permanent teeth and if not, the same treatment as in Case 1 was planned.

## 4. Outcome and Follow-Up

The siblings were advised for periodical follow-ups for prevention of formation of abscesses along with fluoride and pit and fissure sealants application. Regular blood investigations were done to rule out the complications of medications used such as secondary hyperparathyroidism and nephrocalcinosis. On long term follow-up, of about 1 year, they showed improvement in gait physical activity and eruption of permanent teeth.

## 5. Discussion

The term rickets is derived from an old English word “wrickken,” which means “to twist,” and a Greek word “rachitis,” which means inflammation of the spine. When the demineralized osteoid accumulates at the sites of bone formation, bones gradually soften leading to deformities in association with weight-bearing sites.

Initially, environmentally caused rickets was very common; however, when it was treated and prevented by public health measures, the vitamin D-resistant rickets came into picture. Econs and Francis [5] highlighted that some of the patients with rickets did not respond to physiological doses of vitamin D and named the condition as vitamin D-resistant rickets.

Inorganic phosphate plays a major role in many biological systems, including cell membrane functions, energy metabolism, cell signalling, and oxygen transport. In hypophosphatemic rickets, renal proximal tubular resorption is compromised due to mutations in PHEX gene which is a phosphate-regulating gene. It encodes a zinc metalloendopeptidase which is predominantly expressed in osteoblast and odontoblast [3, 5]. So the patient presents with hypophosphatemia along with a relative 1,25-(OH)2 vitamin D deficiency leading to the manifestations of rickets.

Familial hypophosphatemic rickets, in most patients, appears in a familial line of X-linked, dominant inheritance with the same prevalence in both sexes; however, it may also occur sporadically [6, 7]. Hypophosphatemic rickets is identified by clinical and radiological features such as decreased vertical height, craniotabes, rachitic rosary, bowing leg deformity (genu varum) or knock knee (genu valgum), double malleoli sign of ankles, metaphyseal hyperplasia of wrist, and Harrison's groove on the chest [8, 9]. Bowing deformity is common in the femur and tibia. The femur shows the coxa vara type of deformity, in which the neck makes an angle of less than 120° with the shaft of the femur [10].

Hypophosphatemic rickets has been related to several primary and permanent teeth alterations. The most commonly reported ones include normal but thin enamel and globular dentin and enlarged pulp chambers with pulp horns extending up to the dentinoenamel junction [11]. In this report, we did not observe specific enamel defects or hypoplasia in the case, but enamel and dentin layer were quite thin, with highly positioned pulp horns according to the radiographic and clinical evaluation. The aggregation of all these factors contributes to the formation of dental abscesses in cases when enamel is lost due to attrition or incipient caries and pulp infection takes place through poorly calcified dentin leading to multiple periodontal abscesses [12, 13] which is interestingly not found in the present case. The early exfoliation of deciduous teeth in rickets can be attributed to poorly defined lamina dura, dysplastic roots, and hypoplastic alveolar ridge [12]. In the present cases loss of deciduous teeth might have been due to trauma as well in both siblings. Hypophosphatemic rickets may be associated directly with increased prevalence of dental taurodontism [8, 14], an increase in the length of the body at the expense of the root.

Laboratory findings including low serum phosphate concentration and reduced tubular resorption of phosphate corrected for glomerular filtration rate (TmP/GFR) are characteristic. Serum calcium and 25-hydroxy vitamin D are within the normal range or decreased; parathyroid hormone is normal to slightly elevated. Alkaline phosphatase is characteristically elevated. In Case 2, all findings were positive along with low calcium and vitamin D levels whereas in Case 1 there were elevated alkaline phosphatase activity and decreased serum inorganic phosphorus levels. Additionally, the normal physiologic response of hypophosphatemia with supplementation of vitamin D is absent in these siblings suggestive of vitamin D-resistant hypophosphatemic rickets.

A family history of short stature, orthopedic abnormalities, poor dentition, and parental consanguinity may signify inherited rickets and the diagnostic criteria of X-linked hypophosphatemic rickets globally are based on history, clinical examination, and biochemical and radiological evaluation which are significant in these siblings.

The treatment of hypophosphatemic rickets is done by the oral administration of phosphorus and vitamin D. Currently, growth hormone [15] and KRN23 [16] a recombinant human monoclonal antibody targeting FGF23 are under investigation for hypophosphatemic rickets.

In our cases, general management is taking care by using neutral phosphate and calcitriol supplements. Regarding dental complaint Case 1 was given denture (Figure 19) and Case 2 was kept under observation for eruption of permanent teeth.

## 6. Learning Points

- Hypophosphatemic rickets is a rare disorder with dental manifestations seen in spectrum of severity, ranging from very severe, with involvement of nearly the entire dentition, to very mild with normal appearance of the teeth.
- Being dentists, we hardly come across this rare variety of rickets in our clinics. Dental abscess in clinically normal teeth, early spontaneous exfoliation of clinically intact primary teeth, and the numerous teeth missing were the characteristics of patients with hypophosphatemic rickets.
- So early detection, treatment, and proper adherence to treatment provide a good dental future to the patients limiting delayed eruption, spontaneous dental necrosis, and dental diseases classically associated with hypophosphatemic rickets.

## Figures and Tables

**Table 1:** Depicting the laboratory investigations of both the siblings.

Serial number	Investigation	Normal levels	Levels in Case 1	Levels in Case 2
1	Serum inorganic phosphorus	4.5–5.5 mg/dL	3 mg/dL	2.8 mg/dL
2	Serum alkaline phosphatase	100–644 IU/L	1143 IU/L	1378 IU/L
3	Vitamin D	20–50 ng/mL	15 ng/mL	12 ng/mL
4	Serum chloride	98–106 mMol/L	113 mMol/L	89 mMol/L
5	Serum potassium	3.4–5.2 mMol/L	3.2 mMol/L	2.5 mMol/L
6	Serum bicarbonate	19–25 mMol/L	15 mMol/L	11 mMol/L
7	Serum calcium	8.8–10.8 mg/dL	9.1 mg/dL	7.3 mg/dL
